# Structure of Protoplasm

**Published:** 1891-06

**Authors:** Carl Heitzmann


### International Dental Journal.

VOL. XII. JUNE, 1891. NO. 6.

### Original Communications.[1]

### STRUCTURE OF PROTOPLASM.[2]

BY CARL HEITZMANN.

MR. PRESIDENT AND GENTLEMEN OF THE ODONTOLOGICAL SOCIETY. —In the year 1873 I made a discovery of the reticular structure of protoplasm. The discovery, as most of you are aware, consisted of the following facts: I saw in the amœba and other protoplasmic formations scattered granules, and the nucleus suspended in the centre, with powers not higher than about five hundred to six hundred, whereas if I looked at such lumps with powers higher than six hundred,—say eight hundred to one thousand,—I saw from the nucleus emanating conical spokes, which inosculated into the neighboring granules. I found all the granules were interconnected by means of delicate threads, and I observed that the most peripheral granules inosculated with an extremely thin layer enclosing the lump. The nucleus looks granular with low powers in a certain stage of development. In the centre I saw a somewhat larger granule, which is termed nucleolus, and I recognized with higher amplifications fine conical spokes emanating from the nucleolus, inosculating with the coarser granules, and terminating in the

[1] The editor and publishers are not responsible for the views of authors of papers published in this department, nor for any claim to novelty, or otherwise, that may be made by them. No papers will be received for this department that have appeared in any other journal published in the country.

[2] Read before the New York Odontological Society, March, 1891.

wall which encloses the nucleus itself. I said at that time that this reticular structure is by no means seen in all phases of development of protoplasm. I maintained that only in the full stage of development of protoplasm this reticulum is visible. My assertion has been that we see no isolated granules within the protoplasm; but that all the granules are interconnected into what I claimed to be a reticulum.

Necessarily the question arose, What is this reticulum? I have claimed in 1873 that it is the living or contractile matter or substance which produces the granules, the connecting threads, the thin layer ensheathing the lump, and the whole vesicle termed nucleus. I said so because I have seen this reticulum, in a living lump, continually in motion. I could explain by the presence of this reticulum every change in shape, so-called amœboid motion, and in locality, the locomotion. I said that the meshes contained a liquid, which as such could not be living matter, for we know that nothing endowed with properties of life is ever a liquid; but I simply took it to be a nitrogenous liquid being suspended in the meshes, causing the appearance of a honeycomb or sponge.

What I saw at that time, during the changes of place and shape was this: in one spot the protoplasmic lump showed the granules slightly enlarged, put together, as it were, the meshes consequently narrowed, the connecting threads shortened. This condition I called the contraction of the living matter, whereas, on the opposite end the reverse took place. The granules became smaller, the connecting threads finer, the meshes themselves enlarged, and as this went on, both granules and threads became more and more delicate, until at last they disappeared to the eye, even with the best possible lens at my disposal, very much like a rod of glass melted over a flame, being drawn into length, would disappear. This condition I called extension. I concluded that the liquid is driven out from the contracted part to the opposite side. I compared this reticulum with a sponge, whose meshes were saturated with water; but the sponge was enclosed by an extremely thin layer of material,—the same which builds up the trabeculæ of the sponge. For if there were no such layer, the liquid would escape.

I said if I could produce a sponge that would be enclosed with a layer perfectly elastic, extensible to the utmost degree, I could imitate the amœboid as well as the locomotion, by pressing on one end of the sponge, driving the water to the other, which could not escape on account of the enclosing layer.

Gentlemen, this observation was perhaps not made accidentally. My eyes at that time had been educated by many years of careful drawings of microscopical objects. It was certainly not an easy matter for anybody to see that reticulum. Still, I trusted my own eyes; I could demonstrate it to some good microscopists, and in 1873 I published this in a short article in the transactions of the Vienna Academy of Sciences.

At that time I was not aware of one fact, and that is, that five or six years before me, C. Frommann, of Jena, Prussia, had observed a reticulum in some connective-tissue corpuscles. He described a reticular appearance without any illustrations thereof; for what he drew was one single offshoot, emanating from the nucleus, which certainly would not make a reticulum. Still, I mentioned this fact in my book, published in 1883, and was much surprised when I learned afterwards that Frommann had complained of my being unjust towards him, not giving him fully what he deserved. An amicable correspondence ensued between us several years ago, for I was extremely desirous to do that man justice, and I publicly announced that Frommann, in fact, was the first man who saw the reticulum with the higher powers of the microscope. Instead of being thankful to me for this acknowledgment, he once more attacked me. He said that he saw that reticulum with a power not exceeding four hundred and fifty diameters. In the face of this statement, I am at a loss to find out what Frommann had seen at all, for I, with, I believe, expert eyes, was not able to see this structure with the power of four hundred and fifty. I say that not because I claim priority. Claims of priority, you all know, are rather childish. Who first discovered a truth is of entirely secondary consequence. If Frommann claims priority I am perfectly willing to accede it to him; but, nevertheless, I cannot help putting before the public the facts which Frommann himself printed, and, of course, there being no illustrations, only description, we cannot state exactly what Frommann did see, as early as 1867.

The discovery came before the scientific world, and although I was thoroughly convinced of the correctness of the observation, I did not expect contradictions as ardent, as zealous, as bitter as did, in fact, afterwards ensue. I will quote just a few words from different authors to show to you what their views were. Among the Germans it was Friedländer, of Berlin, who saw the reticulum, admitted its presence in the protoplasm, but at the same time claimed that it is caused by reagents, especially by the chromic acid solution, which I have always advocated for preservation of tissues.

Much later, Professor Butschli, of Heidelberg, described a reticular structure, and called it "Honeycombed Appearance of Protoplasma," which is exactly the same as that which I have described.

Among the English authors I wish to quote Alb. E. Schäfer, who, in the second volume of Quain's book on anatomy, gives me credit for the discovery. Further on, E. Klein, of London, my personal friend, who, in the last edition of his book, says that protoplasm is often of reticular structure, especially if treated with certain reagents.

Now, gentlemen, this is not an exact corroboration of what I have said; for, as you will presently see, this assertion is mistaken. I have seen the reticulum in the living protoplasma, and as such it is now photographed in this way.

Far more satisfactory is the description and the picture of Mr. Edward Clodd. He is the author of a scientific work, "The Story of Creation," published in New York, and on page 41 you will see wood-cuts called "Diagram of a cell, showing the honeycomb appearance and the reticulum," just in the same way in which I described it in 1873. Both in the nucleus and in the protoplasma we see the reticulum to perfection. Mr. Clodd does not quote anybody for the discovery, and perhaps he does not know who was the man who first saw it, but, as I said, I would not lay stress upon this. The fact nevertheless stands, that this gentleman knows the structure of a cell as well as I do.

Last year, while in Europe, I entered the laboratory of my dear old teacher, Professor S. Stricker, in Vienna, Austria. I was informed by him that he had been successful in producing, by means of photography, an image which would certainly surprise me. He handed me a volume, on the title-page of which you will find his dedication in German. The title of the volume is "Works from the Vienna Institution of General Pathology, 1890." Here you will see a photograph, not a micro-photograph, which means a small photograph, but a photo-micrograph,—*i.e.*, the photograph of some small object. It is a photo-micrograph of the colorless blood-corpuscle of the proteus that lives in the Adelsberg Cave in Carinthia, Austria. It is without eyes, is peculiar to this cave, and is noted for the large size of its blood-corpuscles. You know we have similar water-lizards or newts in this country, too. This animal served as a topic for the illustration of the colorless blood-corpuscle in question. The so-called electric microscope was used for instantaneous photography. The electric microscope is an apparatus by means of which we can project microscopical specimens with a light not less than forty ampères strong. I am in possession of this apparatus, which I purchased several years ago; but I could not bring it to work, simply because there was no possibility of getting forty ampères light in this city. All the experiments that we have made with the Brush Electric Company, and some other companies, proved to be failures. I could not get a room to put up the electric microscope. I offered it to this great Academy of Medicine and was refused; it seems simply for fear of danger of fire. I said, if they refused it for that reason, I would be willing to pay the surplus of the policy of fire insurance out of my own pocket. Still they would not accept it. Why, is utterly unintelligible to me. Of course, we would have to purchase some more apparatus, a revolving dynamo-machine, which is used for the production of enormous light, and a motor apparatus, which would be an additional expense of eight hundred dollars. I was perfectly willing to incur this expense also if they would give me the room. Nay, I wanted to present it to the Academy, not for the sake of profit, but simply for demonstration. All without avail. Several years ago it looked as though some dentists would take it. It was needed for the demonstration of several delicate observations, and I know that Dr. Bödecker tried hard to bring it to work, but it proved to be an utter failure, simply on account of the lack of intensity of light. Thus the beautiful instrument, which was constructed by Stricker, is still standing in the extension of my house at rest.

The electric microscope enables us to bring forth a power which otherwise could not be reached. Stricker used the power of twenty-five hundred diameters, and, as a result, saw a colorless blood-corpuscle almost two inches in diameter. If you look close, gentlemen, you will see the reticulum that I described in 1873 plainly represented in the photo-micrograph; especially look at the right lower part, where it seems to be in the best focus. You will recognize the granules as glistening lumps, and from these the conical spokes emanating and inosculating into the neighboring granules, thus producing a net-work. At the same time you will see the thin enclosing layer of the protoplasmic lump perfectly plain.

Stricker does not say what the reticulum is. He simply wanted to publish the picture by transferring the photograph by means of heliogravure. It is made without interference of human eye, or human hand, and, you can see, the result is gratifying to the utmost degree. That this reticulum therefore exists, I do not think is any more a topic of discussion or doubt.

The greatest blow that has been made against my views, several years after the publication, was the discovery of karyokinesis, or mitosis. It was Schleicher who did most of the work in the description of the peculiar changes which take place in the nucleus preceding its division. The nucleus, as I described it in 1873, was reticular. New observations were made; the substance of the nucleus appears in the shape of loops, which originally make a wreath, afterwards a star, later a double star, and, at last, lead to the splitting up of the nucleus into what was called its indirect division. Remak, many years ago, described a direct division of the cells, which means that an original cell would become thinner in the centre,—the same being the case with the nucleus,—and there a division would take place, and two individuals arise, by direct division. The karyokinesis led to an indirect division, since it commenced in the nucleus first, afterwards caused a narrowing of the protoplasma, and at last a splitting up into halves. It has been asserted that the formations, being the result of karyokinesis, were, as a rule, lying in a space in no connection with the rest of the protoplasm. I said before, it was the hardest blow on my theory, since I have claimed that the nucleus was nothing but a compact mass of living matter,—whereas the reticulum in the protoplasm was a delicate formation of that same matter. Now, it looked as if the nucleus had nothing to do with the protoplasma. Some authors claimed that it was built up of a material of its own, and they composed the word chromatin, which means a material easily colored by certain dyes, whereas a number of delicate threads visible in the nucleus were called achromatin. Others called the substance building up the nucleus, nucleine; the substance not colored, para-nucleine. Quite a number of words have been established, which, in my opinion, are altogether superfluous. That indirect division exists is out of the question. The question is, What is the substance building up the nucleus? If it were true that we see no connections whatever of the loops of the nucleus with the rest of the protoplasm, then my theory must fall to the ground; there is a perfectly legitimate proof that I was wrong in my assertion that the nucleus and the reticulum are made of the same material.

I have kept quiet about this until quite recently. What I knew was that, if you look into a living, moving protoplasmic lump, you will not see karyokinesis; neither will you see it when you preserve your specimens with chromic acid solutions. Certain peculiar materials are required for bringing forth the loops. The solution of the puzzle is, that somewhat more solid masses of living material will take up certain dyes more easily than delicate formations, whereas others remain unstained, not because they did not take up the colors, but simply because they are too fine to be stained at all, very much in the same way that we have long since known of carmine.

I admitted that often we see a closed space around the karyokinetic figures, but by no means always. I have repeatedly observed connections of these loops by means of conical off-shoots with the reticulum of the surrounding protoplasm, especially in chromic acid specimens, although the karyokinetic figures were not plain. More than that, Schleicher, who was the first to use the word "karyokinesis," directly states that he saw the loops in movement. Now, pray, what substance can there be which is seen directly moving, which must move in order to produce such remarkable figures, and which is in direct connection with the reticulum? Was I right or not? Do we need new Greek words? If we say the nucleus is the same substance which builds up the reticulum of the protoplasm,—viz., living or contractile matter,—we have said everything we need to know. Karyokinesis means movement of the wreath. Mitosis means splitting up to threads. Some others use karyomitosis, but it makes no difference. What I wish to emphasize is that, unless there be present a certain amount of liquid in a vacuole or plasmatic space around the nucleus, the connection of the karyokinetic figures with the rest of the reticulum is visible. Being a member of the American Association of Anatomists, and having examined specimens of such connections, I took them along, last year, to Boston, where we had a meeting, and, with powers of about one thousand, demonstrated the connections with the rest of the protoplasm. At the same time I was disappointed in finding in the work of Klein a statement to the same effect,—viz., that in many instances we see the connections of the mitotic or karyokinetic figures with the reticulum of the protoplasm,—which fact goes far to prove that the substance building up the nucleus is in fact nothing but living or contractile matter.

In looking over the literature of the cells, we are astonished to find so many answers to an apparently simple question: What is the nucleus? Before the discovery of the structure of protoplasm the nucleus had never been considered as something essential. In the original diagram of a "cell" the nucleus played a certain *rôle;* that was in 1839: about fifty years ago. Some thirty years ago, Max Schultze said that there exists no cell-wall at all. In the same way E. Brücke, of Vienna, said the "nucleus is nothing essential, being of secondary importance." Twenty years ago nobody considered the nucleus as necessary to the idea of a cell; and Brücke in 1861, proposed to strike out the granules of the protoplasm too. In this way S. Stricker described protoplasm as a homogeneous, structureless mass, and you see Klein still uses the same wording for its definition. How much we have learned in the last twenty years you will see if you compare the figures of Mr. Clodd. He gives karyokinetic figures, and tries to show structure even in the threads. I cannot follow him in that, since we must admit that living matter is apparently structureless, even to the highest powers, and we cannot analyze it even with the best lenses at our disposal. All we can say is that protoplasm holds a certain amount of living matter, it is not structureless; but as to the structure of the living matter itself we know nothing.

Now, gentlemen, just a year ago, in this very Society, the question of the structure of protoplasm became mooted. You will remember that a resolution was passed that myself and some of my friends should be compelled to demonstrate what we maintained to experts, who would be chosen by this Society. The wording of the resolution was of a kind which plainly showed that it was written by a man who did not know what he was about. He did not know even the elementary nomenclature of our doctrines. Long since the word "protoplasmic" was adopted for the definition of a reticulum built up by protoplasm, and for naming what we call a myxomatous tissue. It is a reticulum made up of protoplasm, in which, of course, there would be visible a reticular structure and scattered nuclei. The reticulum which we call protoplasmic is of a myxomatous nature; but to call the reticulum, which is visible in the protoplasm itself, a protoplasmic reticulum is a great mistake. This mistake was the most formidable weapon against the man who made up the resolution, as you all remember, and Drs. Abbott and Bödecker and I discarded the whole question.

Such are the facts; and now permit me to review what we have done in the last sixteen or seventeen years for the demonstration of the new ideas, especially in the science of dentistry, in dental anatomy. Shortly after my arrival here I was happy enough to become acquainted with a number of excellent men of your profession, who, more by intuition than by conviction, thought that here was something worth looking up. It was, in fact, a remarkable thing that, owing to the kindness, I suppose, mainly of Dr. Wm. H. Atkinson, hundreds of dentists flocked to my laboratory, studying not only dental but also general histology. I could demonstrate to every one of them the presence of the reticulum in the protoplasm, and I suppose every one left the laboratory satisfied that he saw the reticulum. Unfortunately, however, it is not everybody's business to continue such studies. Some told me that after they had left the laboratory, they tried to see the reticulum again, and failed. On the other hand, there was continuous opposition against my assertions, which, as you know, involved entirely novel views concerning the structure of the tissues; and the consequence was that the zeal of the dentists to study dental histology abated in the last six or seven years. Unquestionably every one who is a revolutionist as I am has to stand opposition. Have we ever seen a new idea, contrary to the ruling views, adopted at once? No. Think of old Harvey, who was declared to be an absolute crank for making the assertion that blood flows through the arteries! The old doctrine was that the arteries contained air, and if Harvey announced the idea of a circulation of blood, he was, of course, a crank. Bitter articles were written against him, and it took quite a while, until at last the idea dawned on the minds of some that Harvey, in fact, was right.

I do not claim to be a Harvey. Far from it. But I claim having offered a theory which is far more plausible than the hitherto acknowledged cell-theory. Quite recently I was a witness in a law-suit, where I was cross-examined by Mr. Choate, and this gentleman, among other things, urged me to give an outline of the new views. I saw at once that he tried to make all that ridiculous, as lawyers do; but I quietly told him the following: "Before the Berlin Museum there is put up a grand marble vase, as high almost as this room, colossal in dimensions, but made without taste,—ugly, in fact. I asked my friends there, 'Why could you Berlin people put up such a tasteless concern as this?' The answer was, 'My dear sir, that is one piece of Prussian marble.' 'Oh,' said I, 'here is the new view of the construction of the body.' Imagine that this marble vase was made up of millions, or, as Dr. Sudduth said once, innumerable numbers' of small pieces of mosaic, pasted together by cement, and you would have the old idea of the cell doctrine,—viz., that our body is made up of millions and millions of cells, pasted together by a little intervening cement substance. But if, on the contrary, you say this marble vase is one continuous mass, and although exhibiting different shadings and varieties in structure, nevertheless is one piece, then you have my idea that our organism is one piece,—and there are no isolated cells whatever within the tissues."

Mr. Choate seemed to be well pleased with this comparison, and

I repeat it to you because I think it is a very plain one. What I claim is not absurd; on the contrary, I think it is rather sensible to say that we are one individual, and not made up of millions of individuals. To be sure, in that mass that we call our bodies we have closed spaces or vacuoles in which are circulating detached lumps, very much like in the amœba there are closed spaces or vacuoles in which float detached particles of living matter. In our blood there are floating isolated lumps called red and colorless blood-corpuscles, but they are not a part of our structure. That is not a tissue, for it is liquid.

At the last meeting of the American Society of Anatomists, Professor Gage, of Ithaca, gave us a nice demonstration of the fibrin of the blood, and said the blood is a tissue. I took exception to this view. I said, "There exists no proof that a liquid ever can be a tissue. A liquid cannot be alive. Even the jelly-fish, such as we get in autumn by the handfuls on Coney Island or in the East River, are jelly-like bodies, but not liquids. If the theory that blood is a tissue be correct, then a pailful of sea-water in which there are a hundred fish swimming about is a tissue too, and a droplet of puddle water, in which there are swarming thousands of minute organisms, is likewise a tissue." Professor Gage opposed me, of course. I still maintain that a liquid cannot be alive and cannot be a tissue.

The body, so far as its tissues are concerned, is one continuous mass. There are no isolated cells. There is a continuity of protoplasm all through, and it is the reticulum of living matter that we have traced in all tissues constituting the body, including those building up and forming the teeth. For this is the new view by means of which we can realize that even the enamel and dentine, which are rather hard tissues, must be endowed with properties of life. We see the reaction in the dentine after each filling, and this fact proves that there must be some life. Where is life located? Is it in the lime? Is it in the dentinal fibres? Yes, but is that enough to explain the reaction after each filling, which will cause a hard wall around the filling?

There is something more. It is just the reticulum, the presence of which has been demonstrated in the protoplasm of the pulp, and throughout all hard tissues of the teeth. Its existence was proved in the enamel by Abbott, and quite recently in the dentine by Dr. John I. Hart. William Carr took up this study in my laboratory four years ago, but never could finish it. The conclusive proofs were furnished only in the last few months.

Gentlemen, you will ask me, can I boast of followers in this world, or am I alone in my little school? Ten years ago, Professor S. Stricker, although originally my teacher, adopted my views, and publicly claimed that I was right in my assertion that basis-substance is alive, the same as are the so-called "cells." Virchow, in Berlin, will never admit it. He is the founder of the cellular pathology, and as soon as he admits that there are no cells, his life's work is gone. What he has done remains forever. We will not attack him should he be mistaken. We know too well that our successors will step over our shoulders, and woe to us if they do not treat us and our memory well!

We have all reason to be kind, even with those who are mistaken, providing they do honest work. You may ask, Have I followers in this country, outside of those who have studied in my laboratory? I say yes. Last year a prominent citizen of this city, Mr. Charles F. Cox, delivered a presidential address before the Microscopical Society, which is printed, and which was sent to me by him. I do not know him personally at all.

Permit me to read what he says about me and my views:

"I can well remember, as perhaps you also can, the disgusted incredulity with which this new doctrine was received,—an incredulity in which, I confess, I then shared. I am not sure that the appearance of a reticulum in the prepared blood-corpuscle is even yet generally accepted as evidence of a normal structure of the kind claimed by Dr. Heitzmann; but the claim certainly gains support from the fact that vegetable histologists are pretty well agreed that a more or less similar reticulum is demonstrable in the protoplasm of plants. Professor Goodale seems to have no doubt on this point. . . .

"In the work from which I have just quoted ('Microscopical Morphology,' New York, 1883), Dr. Heitzmann generalizes as follows: 'What . . . was called a structureless, elementary organism, a "cell," I have demonstrated to consist only in part of living matter, while even the minutest granules of this matter are endowed with manifestations of life. The cell of the authors, therefore, is not an elementary, but a rather complicated organism, of which small detached portions will exhibit amœboid motions. . . . How complicated the structure of a minute particle of living matter may be we can hardly imagine; what we do know is that the so-called "cell" is composed of innumerable particles of living matter every one of which is endowed with properties formerly attributed to the cell-organism.'

"It having been shown that life hangs upon a web of infinite tenuity, and does not reside necessarily in either a vesicle or a lump, it was a natural and easy step to extend this net-work from tissue to tissue and organ to organ in an unbroken circuit of vital communication. This step Dr. Heitzmann does not hesitate to take; for, says he, 'there is no such thing as an isolated, individual cell in the tissues, as all cells prove to be joined throughout the organism, thus rendering the body *in toto* an individual. What was formerly thought to be a cell is, in the present view, a node of a reticulum traversing the tissue. . . . The living matter of the tissues exists mainly in the reticular stage, and is interconnected without interruption throughout the body.'

"Again, this at first very strange and, for some reason or other, unwelcome doctrine receives support from the investigations of botanists; for, as Professor Goodale remarks, this protoplasmic intercommunication between adjoining cells 'has been shown to be so widely true in the case of the plants hitherto investigated that the generalization has been ventured on that all the protoplasm throughout the plant is continuous.' The position to which we have traced this matter is, then, that to the latest biology, in any particular organism, a generally diffused and interconnected substance, simple only in appearance under present optical aids, has taken place of the circumscribed, more or less isolated and independent, and recognizably complex vesicle which was the physical basis of life to the science of fifty years ago. In the words of Dr. Heitzmann, 'According to the former view, the body is composed of colonies of amœbæ; according to the latter, the body is composed of one complex amœba.'"

Gentlemen, truth is welcome from whatever quarter it may come. I have had in these last years many dentists in my laboratory, and, of course, I could not help having also opponents, and some of them bitter ones, who in spite of my polite invitations to come and see refused to do so. All I have to do is to heartily thank all who have come to my laboratory, and especially thank those who have done original work there, although their number as yet is rather limited. I have to thank especially Dr. Bödecker, Dr. Abbott, our old friend Dr. Atkinson, and of late Dr. Hart.

Gentlemen, in this country, where practical dentistry is so far ahead, why should we not have more original work in the line of a microscopy? Why should we adhere to the views of those dull fellows over the sea, who do not even do justice to us, as I have shown before the First District Dental Society, and perhaps cannot even realize what we are about? Why should we still depend upon them? We have the means to do original work. Why is it that so few enter it? That we are on the right track I have endeavored to prove to you, and if I have succeeded, I shall feel gratified.

